# B Regulatory Cells: Players in Pregnancy and Early Life

**DOI:** 10.3390/ijms19072099

**Published:** 2018-07-19

**Authors:** Ana Esteve-Solé, Yiyi Luo, Alexandru Vlagea, Ángela Deyà-Martínez, Jordi Yagüe, Ana María Plaza-Martín, Manel Juan, Laia Alsina

**Affiliations:** 1 Functional Unit of Clinical Immunology and Primary Immunodeficiencies, Allergy and Clinical Immunology Department, Hospital Sant Joan de Déu, University of Barcelona, Pediatric Research Institute Sant Joan de Déu, 08950 Barcelona, Spain; a.estevesole@gmail.com (A.E.-S.); yiyibiomedicina@gmail.com (Y.L.); adeya@sjdhospitalbarcelona.org (Á.D.-M.); 2 Functional Unit of Clinical Immunology, Hospital Sant Joan de Déu-Hospital Clínic de Barcelona, Barcelona, Spain; vlagea@clinic.cat (A.V.); jyague@clinic.cat (J.Y.); aplaza@sjdhospitalbarcelona.org (A.M.P.-M.); 3 Immunology Service, Biomedic Diagnostic Center, Hospital Clínic de Barcelona, Universitat de Barcelona, IDIBAPS, 08036 Barcelona, Spain; 4 Allergy and Clinical Immunology Department, Hospital Sant Joan de Déu, University of Barcelona, Pediatric Research Institute Sant Joan de Déu, 08950 Barcelona, Spain

**Keywords:** Breg cells, neonate, pregnancy, cord blood, tolerance

## Abstract

Pregnancy and early infancy represent two very particular immunological states. During pregnancy, the haploidentical fetus and the pregnant women develop tolerance mechanisms to avoid rejection; then, just after birth, the neonatal immune system must modulate the transition from the virtually sterile but haploidentical uterus to a world full of antigens and the rapid microbial colonization of the mucosa. B regulatory (Breg) cells are a recently discovered B cell subset thought to play a pivotal role in different conditions such as chronic infections, autoimmunity, cancer, and transplantation among others in addition to pregnancy. This review focuses on the role of Breg cells in pregnancy and early infancy, two special stages of life in which recent studies have positioned Breg cells as important players.

## 1. The Immune System during Pregnancy and the Neonatal Period

Pregnancy and the neonatal period constitute two special immunological states both for the pregnant women and for the conceptus. On one hand, the mother needs to tolerate the semi-allogenic cells growing in-utero carrying paternal antigens while at the same time needing to be able to deal with infectious microorganisms. As such, it has been proposed that “mammalian pregnancy defies the immune system rules” [1]. Furthermore, the fetus also needs to tolerate the allogenic mother and in particular must transition from this semi-allogenic but virtually sterile maternal uterus to a world full of antigens, including a massive mucosal colonization occurring immediately after birth. Thus, the newborn must develop different strategies to overcome these challenges, including the avoidance of excessive responses, which is critical.

### 1.1. The Immune System during Pregnancy

Pregnancy is not a state of general immunosuppression but rather of local and systemic immune modulation, during which pregnant women have shown increased susceptibility to influenza, measles, hepatitis E, and Herpes Simplex Virus [2]. Tight immune system regulation and modulation is needed, as both inflammatory and anti-inflammatory signals are needed for successful pregnancies [3,4]. In fact, immune-related causes are thought to explain some of the current ‘idiopathic’ causes of infertility. As a result, women with problems in the immune system have greater probabilities of poor pregnancy outcomes [4]. The diverse combination of hormones throughout pregnancy is crucial, since it modulates the recruitment and limits the presence of different innate and adaptive cell types in the maternal-fetal interphase. Concretely, estradiol, progesterone, and human gonadotropic hormone are the main hormonal regulation players, inhibiting destructive immune responses and inducing tolerance-promoting pathways, such as the human gonadotropic hormone-mediated decrease of the cytotoxic capacity of peripheral natural killer cells in pregnant women [1,5,6].

#### 1.1.1. Natural Killer Cells

Natural killer (NK) cells play a critical role during pregnancy, protecting both the mother and the conceptus [3,4,7,8,9]. NK cells represent a high proportion of the decidual leukocytes, and there is an increase of uterine NK cells, characterized by a CD56^bright^CD16^−^ phenotype [10]. Cells with this phenotype have cytotoxic capacities in other tissues, but in the decidua they have pregnancy establishment functions [3,7,11,12]. Concretely, NK cells have an increased relative expression of inhibitory receptors and play an important role in the remodeling of decidual blood vessels [4,13,14] and in trophoblastic migration and invasion [3,13,15]. Furthermore, uterine NK cells interact with human leukocyte antigen (HLA)-G and HLA-C, causing their inhibition [3,8,12]. This is of special interest since the poorly polymorphic non-classical HLA-G is expressed in the extra-villous trophoblast [8].

#### 1.1.2. Myeloid-Derived Cells

Macrophages, dendritic cells (DC), and mast cells play a role in implantation and tissue remodeling. Macrophage population is also regulated during the different phases of pregnancy [1,16,17,18]. Concordantly, there is an increased frequency of M2 macrophages, associated with tissue healing and homeostasis in the decidua [7,16,17,18] in relation to tissue remodeling and angiogenesis [5]. Macrophages are also capable of regulating hormonal levels, as they degrade excessive human gonadotropic hormone [1] and are also responsible for pathogen clearance in the endometrium, therefore showing pro- and anti-inflammatory functions [3]. Interestingly, hormonal changes alter the presence and phenotype of circulating monocytes, which have a role in assisting the implantation of the blastocyst and in pregnancy termination [4]. In addition, DC are mainly maintained at a more immature phenotype with regulatory functions [5]. Mast cells are present in the maternal-fetal interface and favor implantation, angiogenesis, and tissue remodeling and are in a quiescent state until term [3,4,5]; besides, they have a different phenotype compared with classical peripheral mast cells [5].

#### 1.1.3. T Cells

T cells are highly regulated during pregnancy. There is an increase in induced T regulatory cells (Treg), regulated by both hormonal changes and alloantigenic exposure [3,7,19,20]. In fact, Treg cell levels are modulated mainly but not exclusively by interleukin (IL)-10 during the menstrual cycle, creating the needed locally tolerant microenvironment [4,8,20]. A decrease in Treg cell markers has been reported in patients with idiopathic infertility [3]; what is more, Treg cells in the endometrium are capable of recognizing HLA-C [8]. Regarding effector T cells, the reduced T helper (Th)1/Th2 ratio is controversial. Knock out mice for Th-2 related cytokines suggest that the reduction or increase of this ratio is a marker of successful or failing pregnancy but not causative of the final outcome [4,20]. However, accumulation of Th1 cells specific for paternal antigens was associated with insufficient generation of Treg cells and, therefore, caused abortion in a murine model [4,8,21]. On the other hand, gamma-delta T cells are increased during pregnancy and are hormone-regulated, presenting with inhibitory functions [4,8]. Besides, memory cluster of differentiation (CD)8^+^ cells have been found in the fetal-maternal interface with regulatory functions, including CD8^+^FoxP3^+^ cells [20]. Other maternal-fetal tolerance mechanisms include increased IL-10 and transforming growth factor (TGF)-β expression [22], an increase of the inhibitory molecule Program Death-Ligand 1 (PD-L1) in the trophoblastic tissue, the lack of class I and II Major Histocompatibility Complex (MHC) expression required for T cell activation by syncytiotrophoblast cells at the major maternal-conceptus interface [8], and, as reported recently, an increase of B regulatory (Breg) cells [23,24].

### 1.2. The Immune System during the Neonatal Period

Transition from the last stages of pregnancy, through labor, and into the first encounters of the newborn with the real-world requires rapid changes and adaptation of the immune system: from cell autonomous and innate immunity to the adaptive immune system. Although the neonate’s immune system can fight pathogen infections, as a result of the need of tolerance, there is an increased risk for severe infections [25]. During pregnancy, the fetal immune system is active, since the fetus actively generates tolerance to maternal antigens, including specific Treg cells [20]. Immunization through maternal immunoglobulin (Ig)G during late pregnancy is critical, since IgG transfer can confer around three months of broad protection for a variety of infectious diseases including measles, mumps, rubella, and varicella. Altered IgG transfer, observed in very low weight and preterm infants, results in an increased risk of lethal neonatal infection.

The immune system of the newborn has different regulatory mechanisms to promote tolerance. For example, hypoxia during labor can cause tissue damage-enhancing inflammation, making the strong immune bias towards resolution of inflammation and healing very important [26]. Upon Toll-like receptor (TLR)-mediated activation, antigen presenting cells promote Treg cell differentiation; besides, non-inherited maternal antigens challenge also results in CD4^+^ T cell differentiation into Treg cells. Along with Treg cells, myeloid-derived suppressor cells and erythroid suppressor cells are present in cord blood and regulate CD4^+^ T cells, CD8^+^ T cells and NK cell activity. What is more, high adenosine levels in blood after inflammatory events and hypoxic states contribute to the promotion of a tolerogenic state of the immune system through the impairment of MyD88 pathway activation, thus reducing TLR-mediated Th1-polarizing cytokine induction and neutrophil activation [25,27,28,29].

The immune system in the neonatal period shows the following characteristics related to the fact that it is markedly modulated to avoid excessive responses [25,27,28,29,30]: (1) decreased complement system function [31,32]; (2) decreased neutrophil quantity and functions, including respiratory burst [33,34,35,36]; (3) mononuclear presenting cells (monocytes, macrophages, and dendritic cells) have decreased IL-1β, tumor necrosis factor (TNF)-α, and IL-12p70 production, along with normal IL-23 and IL-6 production [37,38,39,40]; (4) decreased Interferon (IFN)-γ production in certain conditions [31,32,41]; (5) interferon responding factor 3 has decreased DNA binding capacity [42]; (6) monocytes produce increased levels of IL-10 and cyclic adenosine monophosphate [37,43]; (7) conventional DC number is decreased and cells are more immature and produce less inflammatory cytokines [44,45], and (8) NK cells have an increased proportion of inhibitory receptors [46,47].

In the neonate, CD4^+^ T-helper cells are biased towards Th2-like immunity, with a more anti-inflammatory profile. However, in response to some insults such as Bacille-Calmette Guerin (BCG) vaccination, the newborn can develop adult-like Th1 responses, and the Th2 bias can be reverted. Besides, Th17 cell levels are low because of reduced transcription of the *RORC* transcription factor gene. There is also impaired T cell signaling by the T cell receptor resulting in decreased transcription of CD40L, IL-12, and IFN-γ-related genes. B cells are mostly naïve with a poor repertoire and diminished B cell receptor activity, resulting in decreased antigen response [25,27,28,29]. Accordingly, newborns have an increased risk for severe invasive infections, specifically intracellular pathogen infections requiring Th1 responses, especially *Listeria monocytogenes*, *Salmonella* spp., and *Mycobacterium* infections [25,27,28].

## 2. B Cells during Pregnancy and Early Life

The role of B cells during pregnancy and early life has been less studied compared to other subsets of the immune system; however, aberrant B cell numbers and functions have been associated with obstetric complications [48]. B cells have been thought of as mere “antibody-factories” over the years; nowadays, it is known that they have other functions including cytokine production and regulation of T cell responses. B cell development and maturation is a complex and regulated process, initiated at 7- to 8-week gestational age in the fetal liver and continued in the bone marrow after gestational age week 17–18 [49,50,51], leading to different B cell subsets in peripheral blood that include naïve, transitional, marginal zone like B-cells (expressing IgM, IgD, and CD27 in their membrane [49,52]), mature B cells, and plasmablasts [49,50]. During pregnancy, to avoid destructive responses, cellular responses are thought to be diminished and compensated for by increased humoral responses [4,8].

### 2.1. B Cells during Pregnancy

Maternal antibody production by B cells during pregnancy has been shown to be both protective and harmful. B cells can produce protective antibodies against paternal antigens, such as asymmetric antibodies that bind paternal antigens but do not produce responses against them. These antibodies are increased by progesterone and gonadotropic hormone [5,20,53,54]. In contrast, immunoglobulin production against infectious agents is critical for immune protection of both the mother and the conceptus [48]. However, besides protective antibodies, auto-antibody production can occur after an infection before or during pregnancy, such as anti-phospholipid antibodies; these can be responsible for pregnancy-associated problems. Indeed, pathogenic antibody production and changes in immune parameters are associated with the appearance of pre-eclampsia [54,55]. Pregnancy hormones also regulate B cell population and antibody production during pregnancy [20,48,54]; their response to mitogens and infectious agents is reduced [48]. Fetal trophoblasts positively regulate the generation of IL-10 producing B cells, related to gonadotropic hormone but not to estrogen or progesterone [4,5,8].

Maternal B cells are reduced throughout the course of pregnancy. There is a reduction in maternal pre-pro and immature B cells observed in bone marrow of pregnant mice during gestation while an increase in mature B cells is observed [56,57]. This modification of the B cell compartment is accompanied by an increase in serum IgA, IgM, and IgG3. These observed changes are hormonally driven, but whether by direct effect or by indirect limitation of the availability of IL-7 remains to be deciphered [57]. Related with these observations, alfa fetoprotein at fetal concentrations can induce B cell apoptosis, thus preventing maternal cells from reaching the fetus [53]. In humans, absolute numbers of B cells in peripheral blood are reduced during the third trimester of pregnancy. Of interest, B cells are present in the amniotic fluid in initial phases of pregnancy [58]; additionally, there is an increased frequency of naïve B cells and a reduction in the frequency of transitional and Breg cells. The selective reduction of Breg and transitional B-cell in peripheral blood may be caused by a migration to the uterus, although this has not been confirmed [59].

### 2.2. B Cells in the Neonatal Period

Neonatal B cells are associated with tolerance and inhibitory mechanisms. It is known that infusion of stem cells from cord blood, rather than adult bone marrow, enables transplantation in patients with increased donor-recipient HLA-mismatch [60], and one of the possible mechanisms explaining this augmented allogenic tolerance is B cell-mediated regulation through Breg cells [61]. Because of maternal antibodies and B cell immaturity, not all vaccines are successful when given at birth, as is the case with oral polio, measles, and rubella vaccination [25,27,28,29]. A few published studies on B cells in the neonate have associated B cells with the Th2 bias: asthmatic mothers of infants with early-allergy had an increase in transitional B cells in the late-pregnancy period, in contrast to non-asthmatic mothers, suggesting that these cells could have a role in the Th1/Th2 bias observed in neonates, which might justify the food allergy [59,62]. B cells [63], and more concretely IL-10 production by B cells [64], have been shown to be important in inflammation in mice. Significantly, murine studies have shown that CD5^+^ B cells in newborn mice also contribute to a decrease in IL-12p70 production [65]. However, it was not until 2010, thanks to Blair et al., that the first description of the phenotype and function of a human Breg cell subset was published [66]. 

## 3. The Breg Subpopulation and Its Role in Health and Disease

Breg cells are a rare B cell subpopulation with regulatory/suppressor functions, and they are one of the peripheral tolerance mechanisms. Breg cells represent less than 10% of total B-cells in circulation and their regulatory activity is mostly but not uniquely performed through IL-10 production. Nevertheless, less than 20% of cells from the different described subsets are IL-10 producers after stimulation [67,68]. There are two theories regarding Breg development: the first states that B cells are a specific lineage with a specific transcription factor that controls the suppressive nature of the cells, while the second suggests that B cells can take on a regulatory phenotype after certain stimuli to suppress inflammation. Inflammation is a potent trigger of Breg cell development and differentiation; Breg cells need a combination of different molecules to become activated, including TLRs, CD40, the B cell receptor, CD80, CD86, and cytokines [67,68].

Currently, there are eight approaches to defining Breg cells in humans [67,68,69,70]; indeed, several markers have been used for the detection and sorting of Breg cells, but as yet, there is no consensus regarding which markers should be used. The different approaches are summarized in Table 1.

The main functions of Breg cells include inhibition of Th1 cells activation, Th17 differentiation and promotion, and maintenance of the Treg cell population [67,68,69]. The major suppressive mechanism for Breg cell function is IL-10 secretion. IL-10 is a suppressor cytokine that can inhibit chemokine and pro-inflammatory cytokine production, thereby inhibiting the effector mechanisms of the immune system. An IL-10 blockade partially inhibits their regulatory function. Although IL-10 is a key player in Breg inhibition of inflammation, other mechanisms have been described. These mechanisms include TGF-β (especially for the differentiation of tolerogenic DCs) and indoleamine 2,3-dioxygenase (IDO) production, cell-to-cell contact by CD80/86 interaction with T cells, PD-L1 inhibition of T follicular helper cells, and CD73-dependent adenosine production [66,68,70,71,75,82,83,84].

The most studied subset of Breg cells is defined by CD24^hi^ and CD38^hi^ expression in B-cells [66,71,85]. Phenotypically, these cells also express IgM, IgD, CD5, CD10, and CD1d [66], resembling transitional B cells [86]. Breg-cells are mainly defined by their regulatory function: Mauri et al. demonstrated that the CD19^+^CD24^hi^CD38^hi^ subset is enriched in IL-10 production and can inhibit IFN-γ production [66,87] and block Th1 and Th17 differentiation while maintaining the Treg cell population [71]. Their implication in human immune-related diseases has mostly been studied in autoimmune and allergic diseases [88,89,90,91,92,93,94,95,96,97], persistent infections such as human immunodeficiency virus (HIV) [72], hepatitis B virus (HBV) [98], *Mycobacterium tuberculosis* [99], cancer [100,101,102,103], transplantation [87,104,105,106,107], and, as demonstrated recently, pregnancy [19,23,24,59,62,88,108].

Viruses, bacteria, helminths [109], and parasites [110] can imbue B cells with regulatory functions. Rapidly after infection, *Salmonella* and *Listeria* induce the apparition of IL-10-producing cells in a TLR/MyD88-dependent fashion in mice. Some helminth-derived molecules can directly promote IL-10 stimulation in murine B cells. These B cells can suppress immune responses towards allergens; studies in humans have also shown this bystander regulatory function after helminthic infections. The lack of helminth infections in westernized countries has been proposed as one of the reasons for the increased incidence of allergy and autoimmunity. The role of Breg cells in viral infection has been more studied because of the part they play in HIV infection and in chronic HBV infection [109]. Immature Breg cells inhibit IFN-γ production by CD8^+^ T cells after HBV virus infection [67,98]. Furthermore, CD24^hi^CD38^hi^ IL-10-producing cell frequency directly correlates with HIV virus load [111]; furthermore, after in vitro Breg depletion, CD8^+^ T cell effector function is restored and HIV infected CD4^+^ cells are cleared in vitro [72].

## 4. Breg Cells in Pregnancy

**Figure 1:**
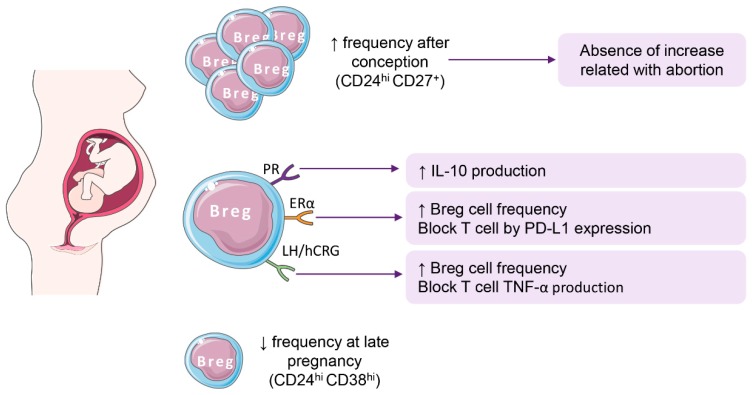
Breg cells in pregnancy. Breg cells are modulated throughout pregnancy, with a major role in pregnancy establishment. Pregnancy-related hormones play a major role in regulating Breg cell frequency and functions. PR: progesterone receptor, ER: estrogen receptor; LH/hCRG: human chorionic gonadotropin receptor.

Breg cells are believed to promote a stable tolerant immune profile in the local microenvironment. Recently, Guzman-Genuino et al. reviewed how the previous knowledge of the role of Breg cells in autoimmunity and transplantation (promoting tolerance) and cancer (promoting tumor growth) could help in the understanding of the role of Breg cells in the establishment and maintenance of pregnancy, where a semi-allogenic mass of cells grows inside a woman [19]. Immunological changes are needed to avoid allogenic reactions that could lead to miscarriage. Body conditions to allow conception and implantation, as well as changes needed to allow embryonic and fetal growth, are regulated by pregnancy hormones. Pregnancy hormones modify immune responses after conception, including Breg cells (Figure 1). Gonadotropic hormone increase, a CD1d^high^CD5^+^ IL-10-producing Breg cell subset, and PD-L1 expression in B cells protect mice from experimental autoimmune encephalomyelitis [19,23,112,113,114,115,116]. Progesterone promotes Th2-like immune responses with a reduction in pro-inflammatory cytokines and increased IL-10 production that is associated with B cell expansion. Human gonadotropic hormone from pregnant women sera increase IL-10 production by B-cells [23,24].

The association of Breg cells with pregnancy success were first observed in mice. In pregnant mice, the increase in CD5^+^CD1d^+^ Breg cells is necessary to avoid immunological abortion. In fact, the transfer of Breg cells to abort-prone mice promotes fetal-maternal tolerance by leading to a Treg cell increase and by maintaining DCs in an immature state [108]. In humans, it was observed that women treated during pregnancy with rituximab, a B cell-depleting antibody, presented a higher rate of first-trimester pregnancy loss [117]. A recent review on the effects of rituximab treatment in patients with autoimmune dysregulation within 6 months of conception revealed that patients had a 12% rate of spontaneous abortion, with 40% reported delivery before 37 weeks, and also 39% of newborns had low B cells counts. On the other hand, at 6 months, B cell counts were normalized and infants did not show any clinical adverse effects. These results should be interpreted with caution due to the coexistent use of other immunomodulatory drugs, the effect of maternal condition, and the need for a longer follow-up of the exposed infants [118], but overall, they suggest the important role of B-cells in pregnancy.

CD24^hi^CD38^hi^ [66] and CD24^hi^CD27^+^ [75] have recently been used for the study of Bregs in pregnant women [23,59]. CD24^hi^CD27^hi^ cells increase in the first trimester of pregnancy but, as observed with murine Breg cells, this increase does not occur in patients who miscarry (Figure 1). In addition, almost 95% of CD24^hi^CD27^hi^ B cells express the receptor for human gonadotropic hormone [24]. These data highlight the importance of B-cells, specifically Breg, in the mother’s achievement of immune tolerance during the first stages of pregnancy. The importance of this subset of cells in the fetus and the newborn is still to be determined. It has recently been reported that maternal B cells are necessary for the development of perinatal tolerance after mucosal antigen application in a murine model, to protect progeny from experimental allergic airway inflammation. In fact, in the absence of B cells there is an aggravation of the allergic response in the progeny. This tolerance has been related to forkhead box P3 (FoxP3) antigen priming by the IgG contained in the amniotic fluid. However, these observations were made in a non-physiological model; interpretation needs to be made with caution [119]. Moreover, Breg cells (CD24^hi^CD38^hi^) have been shown to be reduced during late pregnancy compared with non-pregnant women [59] (Figure 1). 

## 5. Breg Cells in Early Life

**Figure 2:**
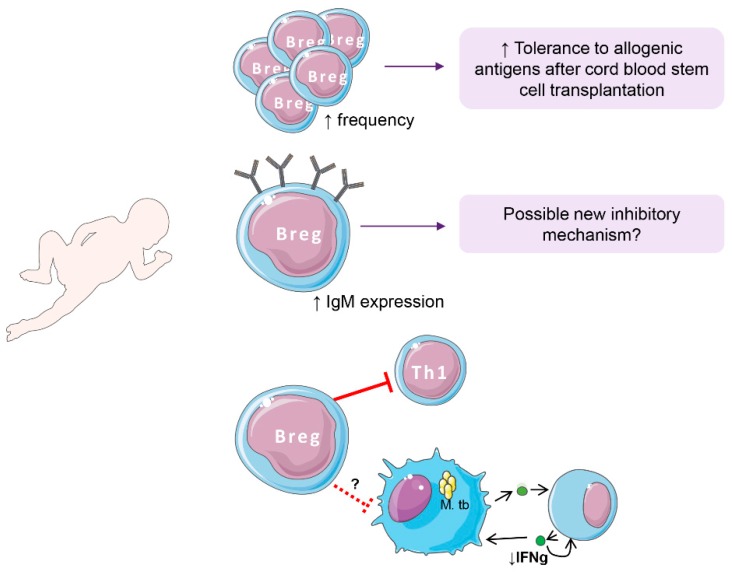
Breg cells in the neonate. Breg cell frequency is increased in neonates, providing improved tolerance to allogenic antigens (i.e., after cord blood stem cell transplantation). Besides, there is a remarkable increase of IgM expression, which may be a new inhibitory mechanism of Bregs in neonates. Finally, Breg cells inhibit Th1 cell responses, which may be related to the reported low interferon (IFN)-γ production after mycobacterial challenge. M. tb: *Mycobacterium tuberculosis*, Th1: T helper 1.

CD24^hi^CD38^hi^ B cells have recently been shown to be at an increased frequency in cord blood of healthy neonates (Figure 2). This B cell subpopulation showed regulatory capacities with a phenotype similar to adult CD24^hi^CD38^hi^ cells and were preventive of IFN-γ production by T cells when co-cultured [61,120]. Of note, Breg cell frequency has been associated with IFN-γ production after whole blood BCG-stimulation (Esteve-Solé et al., unpublished data). Increased frequency of CD24^hi^CD38^hi^ B cells in neonates could be ascribed to the immaturity of the system, these cells being ‘only’ transitional B-cells; however, functional studies have confirmed that neonatal CD24^hi^CD38^hi^ B cells have regulatory functions. Neonatal CD24^hi^CD38^hi^ B cells produce IL-10 upon stimulation, having a suppressive activity on IFN-γ and IL-4 production by T cells [61,120]. Furthermore, neonatal CD24^hi^CD38^hi^ B cells have a similar phenotype compared with adult CD24^hi^CD38^hi^ Breg cells [66,67,71], with increased IgM and decreased CD27 expression. Since CD27 is a memory marker, the decrease can be explained by their very young age [66]. In addition, the presence of elevated levels of IL-10 producing CD24^hi^CD38^hi^ cells has been associated with good outcomes in neonatal late-onset sepsis [121].

IL-10 blockade experiments performed in neonatal Breg cells revealed that part of their function can be carried out via IL-10-independent mechanisms [61]. Immune regulation by cord blood B-cells seems to be partially mediated by (i) IL-10 production and (ii) cell-to-cell direct contact (mediated by CD80/CD86), but independent of TGF-β, as has also been observed in adults [61,67]. On the other hand, the increased quantity of IgM per cell in neonatal Breg cells compared both to non-Breg (cells with low expression of CD38 and CD24) cells and adult Breg cells [120] could be a new mechanism of action for their inhibitory function (Figure 2). Naturally occurring IgM anti-leukocyte autoantibodies (IgM-ALA) have a suppressor capacity that can inhibit T cell activation and chemotaxis [122,123]. IgM-ALA antibodies are present at birth [123], and they inhibit proinflammatory cells from producing IFN-γ and IL-17 in response to alloantigens in mice [122]. More research should be done to evaluate whether this is a true regulatory mechanism of neonatal Breg function.

One of the studies on neonatal Breg cells revealed an increase in these cells in the neonatal marginal zone-like B cell subset [120]. Circulating marginal zone B cells are representative of splenic marginal zone B cells; these cells are characterized by a pre-diversified Ig repertoire and by initiating T cell-independent responses through TLRs as activation signals. Their responses are mainly directed against encapsulated bacteria, including commensal microbiota [52,124,125], and they play a role in normal pregnancy development [126]. As TLRs activate Bregs [66,75], the greater proportion of marginal zone-like B cells observed may indicate increased regulatory responses after encapsulated bacterial stimulation, thus explaining lower responses to encapsulated bacteria in infants [30]. This could be of importance in the rapid abrogation of unwanted responses to commensal bacteria. It may be hypothesized that during the first contacts with the extra-uterine environment and the adoption of microbiota, the increased proportion of Breg-cells among marginal zone B cells is one of the mechanisms by which the neonatal immune system protects itself from an exacerbated response to the new range of antigens encountered.

Increased Breg levels in umbilical cord blood have been related to lower rates of graft versus host disease (GvHD) in cord-blood transplanted patients [61,127] (Figure 2). In addition, patients developing GvHD present a decreased frequency of Breg cells [61]. However, cord-blood transplantation has also been associated with an increased morbi-mortality due to infections [127]. We hypothesized that, as CD5^+^ murine B-cells contribute to the reduced production of IL-12 by antigen presenting cells [128], increased neonatal Breg cell subset contributes to the limited Th1 response observed in neonates inhibiting IFN-γ production. Furthermore, the frequency of Breg cells in the neonate can predict the severity of acute bronchiolitis disease after respiratory syncytial virus, thus showing how neonatal Breg cells can modulate microbial pathogenesis [129]. In our group, we recently observed an inverse correlation between the frequency of Breg cells and IFN-γ secretion after whole blood Bacille-Calmette Guerin (BCG) stimulation (unpublished data). It is known that after BCG challenge in neonates, the major providers of IFN-γ are NK cells instead of T cells [130]; therefore, Breg cells could be, at least in part, responsible for this decreased T cell response. This association, together with the fact that high IFN-γ levels are associated with graft versus host disease development [131,132], deserves further investigation and may have implications in clinical practice, especially in stem cell transplantation from cord blood. For example, in IFN-γ receptor 1 deficiency, where baseline levels of IFN-γ in blood are increased [133,134], stem cell transplantation has been difficult to perform with success [135]. We propose that umbilical cord blood transplantation in these patients could help to reduce IFN-γ levels in blood, thus helping the engraftment (Figure 2).

## 6. Conclusions

B cells in the hallmark of pregnancy and early life have been understudied. However, in recent years there has been growing interest in this lymphocyte population, especially the recently discovered Breg cell subset. Breg cells seem to be an important player permitting pregnancy establishment. The observation of an expanded Breg cell subset in cord blood opens the door for new research and possible treatments, including the study of new mechanisms of action, such as (i) the possible role of the highly increased levels of IgM in neonatal Breg cells in the inhibitory mechanisms, (ii) the definition of the role of Breg in different conditions of health and disease, such as neonatal infection, autoimmunity, and inflammation, as well as the effect of maternal diseases on the Breg cell population in the neonate, and (iii) the evolution of this subset during the first years of life and its relation to childhood immune-mediated diseases. Also, it is advisable to better characterize the role of Breg cells in cord blood transplantation, where they may act as beneficial actors in achieving tolerance or as detrimental actors in susceptibility to infection.

The establishment of pregnancy, its maintenance, and the peripartum period involve complex states, tightly regulated by intricate relationships among the different cell subsets of the immune system. In light of recent discoveries about the Breg cell subset in these situations, there is a need to consider this subset in the overall scheme. However, since few studies have been carried out, more in-depth research must be done to clarify the role of Breg cells and their interaction with their immune system counterparts to reveal the relationships among them and the other generators of tolerance in pregnancy and early life.

## Figures and Tables

**Table 1:** Described B regulatory (Breg) cell subsets. DC = dendritic cells; NK = natural killer; IL = interleukin; PD-L = program death-ligand; Ig = immunoglobulin; TIM1 = T cell immunoglobulin and mucin domain; TGF = transforming growth factor.

Name	Phenotypic Markers	Function	References
Immature B-cells	CD24^hi^CD38^hi^	perform their action on CD4 and CD8 T cells, plasmacitoid DCs, and invariant NK T cells by IL-10 secretion, and PD-L1, CD80, CD86, and CD1d ligation	[66,71,72,73,74]
B10 cells	CD24^hi^CD27^hi^	produce IL-10 and regulate monocytes and effector CD4 T cells	[75]
Granzyme B^+^ (GZMB) cells	CD38^+^CD1d^+^IgM^+^CD147^+^	regulatory function on CD4 T cells by IL10, indoleamine 2,3-dioxygenase (IDO), and GZMB	[76]
Br1	CD25^hi^CD71^hi^CD73^low^	produce IL-10 and allergen-specific IgG4, thus suppressing allergen-specific CD4 cells and maintaining allergen tolerance	[77]
Plasmablasts	CD27^int^CD38^hi^	produce IL-10, but their target cell type is not known yet	[78]
-	CD39^+^CD73^+^	regulate CD4 and CD8 T cells by adenosine formation, thus reducing inflammation by adenosine triphosphate	[79]
Induced Breg cells	-	produce TGF-ß and IDO to suppress CD4 T cells. These cells are developed after T cell cytotoxic T-lymphocyte antigen 4 interaction	[80]
-	Express TIM1	inhibit CD8 and CD4 T cells by producing IL-10	[81]

## Abbreviations

BCG	Bacille-Calmette Guerin
Breg	B regulatory
CD	Cluster of diferntiation
DC	Dendritic cell
FoxP3	Forkhead box P3
GvHD	Graft versus host disease
GZMB	Granzyme B
HBV	Hepatitis B virus
HIV	Human immunodeficiency virus
HLA	Humanl leukocyte antigen
IDO	Indoleamine 2,3-dioxygenase
IFN	Interferon
Ig	Immunoglobulin
IgM-ALA	Igm anti-leukocyte autoantibodies
IL	Interleukin
MHC	Major histocompatibility complex
NK	Natural killer
PD-L	Program death-ligand
TGF	Transforming growth factor
Th	T helper
TIM	T cell immunoglobulin and mucin domain
TLR	Toll-like receptor
TNF	Tumor necrosis factor
Treg	T regulatory
